# Transcriptional Regulation of Cell Cycle Genes in Response to Abiotic Stresses Correlates with Dynamic Changes in Histone Modifications in Maize

**DOI:** 10.1371/journal.pone.0106070

**Published:** 2014-08-29

**Authors:** Lin Zhao, Pu Wang, Haoli Hou, Hao Zhang, Yapei Wang, Shihan Yan, Yan Huang, Hui Li, Junjun Tan, Ao Hu, Fei Gao, Qi Zhang, Yingnan Li, Hong Zhou, Wei Zhang, Lijia Li

**Affiliations:** 1 State Key Laboratory of Hybrid Rice, College of Life Sciences, Wuhan University, Wuhan, China; 2 Renmin Hospital, Wuhan University, Wuhan, China; Peking University Health Science Center, China

## Abstract

The histone modification level has been shown to be related with gene activation and repression in stress-responsive process, but there is little information on the relationship between histone modification and cell cycle gene expression responsive to environmental cues. In this study, the function of histone modifications in mediating the transcriptional regulation of cell cycle genes under various types of stress was investigated in maize (*Zea mays L.*). Abiotic stresses all inhibit the growth of maize seedlings, and induce total acetylation level increase compared with the control group in maize roots. The positive and negative regulation of the expression of some cell cycle genes leads to perturbation of cell cycle progression in response to abiotic stresses. Chromatin immunoprecipitation analysis reveals that dynamic histone acetylation change in the promoter region of cell cycle genes is involved in the control of gene expression in response to external stress and different cell cycle genes have their own characteristic patterns for histone acetylation. The data also showed that the combinations of hyperacetylation and hypoacetylation states of specific lysine sites on the H3 and H4 tails on the promoter regions of cell cycle genes regulate specific cell cycle gene expression under abiotic stress conditions, thus resulting in prolonged cell cycle duration and an inhibitory effect on growth and development in maize seedlings.

## Introduction

Plants respond to unfavorable environmental conditions such as cold, heat, drought, high salinity and heavy metal. These stresses suppress the plant growth and development, which is shown to be a consequence of inhibition of the cell proliferation as well as the cell expansion [1]–[3]. The cell proliferation is regulated by the cell cycle that is composed of a series of phases and progression of eukarytotic cell cycle and is primarily controlled by a class of heterodimeric protein kinases consisting of a catalytic subunit known as a cyclin-dependent kinase (CDK) and a regulatory subunit, cyclin [4], [5]. The catalytic activity of CDKs requires cyclin binding and activation and is modulated by different mechanisms [6]. A significant number of genes which regulate the cell cycle have been quite well characterized in plants. Plant cyclins have been classified as of types A, B, C, D, H, P and T. These types of cyclins were identified in plants, mainly by analogy to their human counterparts [7]. Several evidences showed that cell cycle regulation plays an important role in growth responses under unfavorable conditions. In wheat (*Triticum aestivum*), a reduction in size of the leaf basal meristem is associated with reduced CDKA activity [8]. In Arabidopsis (*Arabidopsis thaliana*) roots, mild salt stress leads to loss of CDK activity and to reduced promoter activity of *CYCB1;2* [9]. Severe salt stress transiently decreases the expression levels of the cyclins *CYCA2;1* and *CYCB1;1* [10].

A variety of histone modifications have been identified, including acetylation, methylation, phosphorylation, and ubiquitination [11], [12]. These histone modifications affect gene expression by altering chromatin structure and accessibility of transcription factors [13]. Histone hyperacetylation is generally associated with transcriptionally active chromatin, while the deacetylated histone is always located on inactive chromatin regions [14], [15]. Recent studies revealed that the changes of histone modification occur under abiotic stresses in plants. In *Arabidopsis thaliana*, modifications on sites of histones H3 and H4 correlate with transcriptional activities of several genes involved in development, flowering, transposon repression and abiotic stress response [16]–[19]. The histone deacetylation and methylation were reported to be associated with repression of *FLC* gene expression during vernalization in Arabidopsis [20], [21]. Previously, research revealed that hyperacetylated histones H3K9 and H4K5 at the gene promoter region are necessary for the osmotic stress-induced transcription of the *ZmDREB2A* gene [22]. Similarly, histone deacetylase inhibitor trichostatin A (TSA) selectively suppresses the induction of cold-responsive transcription factor gene *ZmDREB1* through elevating histone modification levels and remodeling chromatin in the promoter region [23]. And cold-mediated unsilencing of heterochromatic tandem-repeated sequences is also accompanied with epigenetic regulation [24].

Numerous studies have demonstrated that chromatin regulation is involved in the expression of the stress-associated genes. However, little information regarding the relationship between histone modification and cell cycle gene expression under abiotic stress in plants was reported. In this study, the results show that abiotic stresses lead to perturbation of cell cycle progression and inhibition of growth and development in maize seedlings. Therefore, the expression of *CDKA* and five identified cell cycle genes (*CycA1;1*, *CycB1;2*, *CycD2;1*, *CycD5;1* and *CycD5;2*) exposed to control and stress conditions was compared. Finally, the patterns of histone modifications H3ac, H3K9ac, H4K5ac and H3K4me2 in maize cell cycle genes in response to various environmental stresses were analyzed. These results demonstrated that dynamic chromatin regulation occurs in response to abiotic stresses on the promoter regions of cell cycle genes and provide a link between histone modification and expression of specific cell cycle genes under unfavorable conditions.

## Materials and Methods

### Plant Materials and Stress Treatment

Seeds of maize (Zea mays) Huayu 5 were germinated and the seedlings were grown in water under 16 h light (120±10 µmol m^−2^ s^−1^) and 8 h dark regime. To initiate stress treatment, 3-day old seedlings were divided into six groups, two of which were respectively transferred to 4°C and 42°C incubators. Other four groups in a 25°C incubator were separately supplemented with 250 mM mannitol, 200 mM NaCl, 200 µM CuSO_4_ and water. After culture for 24 h, all samples were harvested. These treatments did not cause maize seedlings premature senescence.

### Growth Analysis

To calculate maize root length, images of seedlings were captured. The root length was quantified using the ImageJ software (NIH, Bethesda, MD, USA). After the root fresh weight was measured, plant samples were dried at 70°C for 72 h; then the root dry weight was recorded. To measure the chlorophyll content, the maize leaves were cut into segments of 10 mm from three individual plants per treatment. They were placed in aqueous 80% acetone and stored for 24 h in darkness. Each chlorophyll concentration was determined spectrophotometrically by measuring the extinction of the extract at the major red absorption (QY) maxima of Chlorophyll a (∼663 nm) and b (∼645 nm) [25].

Ca (mg/g) = [12.7×A663–2.69×A645]×V/1000×W (Chlorophyll a)

Cb (mg/g) = [22.9×A645–4.86×A663]×V/1000×W (Chlorophyll b)

Where V = volume of the extract (ml); W = Weight of fresh leaves (g).

### Paraffin wax sections

Representative root samples from differential stresses were removed and put in FAA solution [glacial acetic acid, formalin (37%) and ethanol (70%); 1∶2∶17 (v/v)] for approximately 24 h at room temperature. The fixed root samples were processed in a Leica TP-1050 tissue processor (Leica Instruments, Oberkochen, Germany) to achieve critical drying and wax infiltration. Serial tissue sections (thickness: 8 mm) from chilled wax-embedded tissues were obtained using a rotary microtome and floated on the surface of a water bath (approx. 42°C) in order to flatten the wax sections. The sections were placed on slides by putting the slide beneath the wax sections and lifting upwards from the water bath. The plant tissues were affixed to the slide surface by melting the wax on hot plate (approx. 60°C). The slides were then oven-dried at approximately 38°C, passed through a schedule of de-waxing, staining and mounting [26]. The prepared slides were observed through an Olympus BX-60 light microscope (Olympus, Tokyo, Japan) and photographed using a CCD monochrome camera Sensys 1401E (Photometrics, Tucson, AZ, USA).

### Western Blot Analysis

Proteins were extracted from the seedlings by grinding the samples in liquid nitrogen and resuspended in the extraction buffer [100 mM Tris-HCl pH 7.4, 50 mM NaCl, 5 mM EDTA and 1 mM PMSF]. The amount of protein extracts was quantified using Bradford assay and further evaluated by visual checking of the Coomassie Brilliant Blue–stained SDS-PAGE. Western blot detection was carried out as previously described [27], [28].

### Immunostaining

Nuclear isolation and immunostaining were performed based on the method described by Hu et al [23]. The root samples were chopped in nuclei extraction buffer (10 mM MgSO_4_, 50 mM KCl, 5 mM Hepes, 1 mg/ml DTT and 0.2% Triton X-100). After filtering through a 33-µm nylon mesh and centrifugation under 200 g for 10 min at 4°C, nuclei were resuspended in the extraction buffer. Next, the resuspended nuclei were fixed in 4% paraformaldehyde for 30 min and then spread on slides. Slides were incubated with the primary antibody at 4°C overnight and incubated at 37°C for 2 h with the secondary antibody. Primary antibodies were diluted 1: 100 in 1% BSA dissolved in 1×PBS. Secondary antibodies were conjugated to FITC. DNA was counterstained with 0.2 µg/ml DAPI (Sigma, MO, USA). Images captured with a CCD monochrome camera Sensys 1401E under an Olympus BX-60 fluorescence microscope (Olympus, Tokyo, Japan), and were pseudo-colored using the software MetaMorph 7.7.2 (Universal Imaging Corp, Downingtown, PA, USA). The microscope settings and exposure times were kept constant for each respective channel.

### Flow Cytometry

Nuclear isolation was performed according to the previously described method [23]. The samples were chopped with nucleus isolation buffer [10 mM MgSO_4_, 50 mM KCl, 5 mM Hepes, 1 mg ml^−1^ dithiothreitol (Sigma, St. Louis, MO) and 0.2% Triton X-100] and filtered through a 33 µm nylon mesh. The nuclei were fixed in 4% paraformaldehyde for 30 min, then were precipitated (200 g, 10 min, 4°C) and resuspended in the isolation buffer.

### Reverse transcription and quantitative real-time PCR

Total RNA was isolated with TRIzol reagent (Invitrogen, Carlsbad, CA, USA) and first-strand cDNA was synthesized from 1 µg of total RNA by using RevertAid First Strand cDNA Synthesis Kit (Fermentas, Burlington, ON, Canada) according to the manufacturer's instructions. Primers were designed using the Primer premier 5 software (melting temperature  = 60°C±1°C; amplicon length, 120–200 bp) (Table S1). Quantitative real-time PCR was performed using SYBR Green Real-time PCR Master Mix (Toyobo, Tokyo, Japan) on the ABI StepOnePlus real-time PCR systems (Applied Biosystems, Foster City, CA, USA). The PCR amplification reactions were 95°C for 2 min, 40 cycles of 5 sec at 95°C, 56°C for 15 sec and 72°C for 20 sec. Relative expression levels were calculated using the 2^−△△Ct^ method [23] and were normalized with the *actin* gene. Experiments were performed in triplicate biological replicates.

### Chromatin immunoprecipitation (ChIP) assay

ChIP assay was performed according to the previously validated method [22], [29]. Soluble chromatin was incubated with specific antibodies overnight at 4°C. The antibodies specific for histone modifications analyzed in western blot and ChIPs were purchased from Millipore (Millipore, Billerica, MA, USA): anti-H3K9ac (07-352) Anti-H3ac (06-599) and anti-H3K4me2 (07-030). Anti-H4K5ac (ab51997) and anti-H3 (ab1791) were purchased from Abcam (Abcam, Cambridge, MA, USA). Mock immunoprecipitation using rabbit serum was performed as a negative control. DNA was purified by phenol/chloroform extraction and ethanol precipitation and used as the template for quantitative real-time PCR with the primer sets for ChIP-PCR assay (Table S2).

## Results

### Various abiotic stresses inhibit maize root elongation

**Figure 1:**
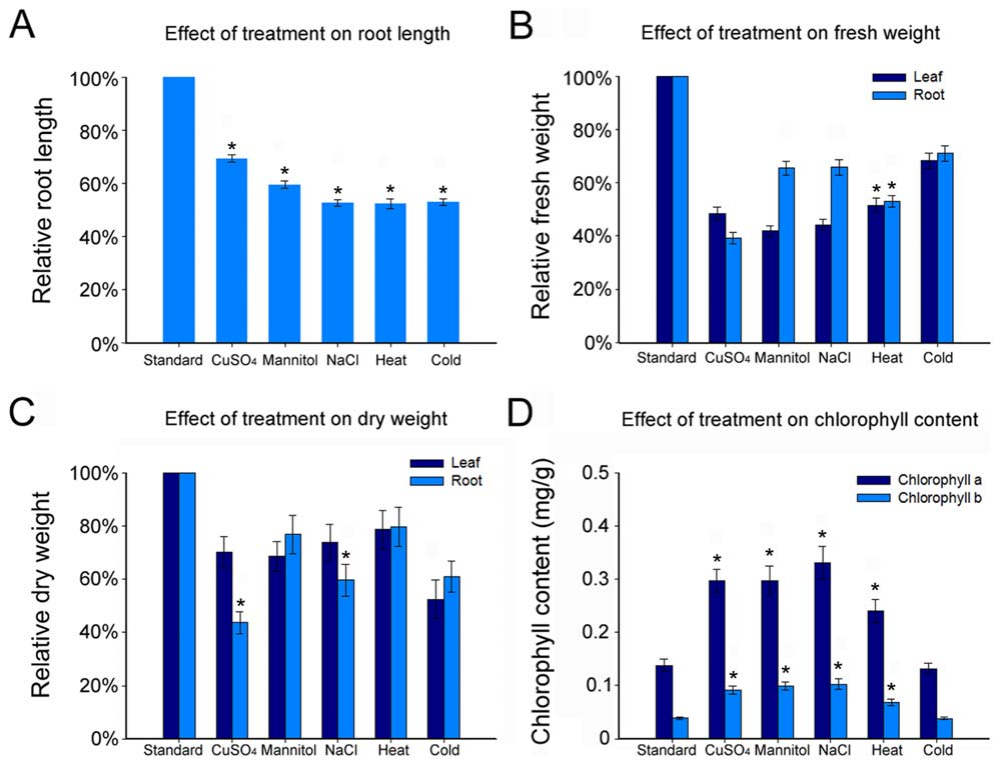
Effects of Various types of stress on the growth of maize. (A) Average primary root length for seedlings transferred to different treatments. Measurements are expressed as a percentage of the length under standard conditions (n>100 seedlings). (B) Average fresh weight for seedlings transferred to different treatments. Measurements are expressed as a percentage of the weight under standard conditions (n>100 seedlings). (C) Average dry weight for seedlings transferred to different treatments. Measurements are expressed as a percentage of the weight under standard conditions (n>100 seedlings). (D) Chlorophyll a and chlorophyll b contents are measured under different abiotic stresses relative to standard condition in maize seedlings. Bars indicate Standard Error (SE). * *p*<0.05 as compared with standard group.

**Figure 2:**
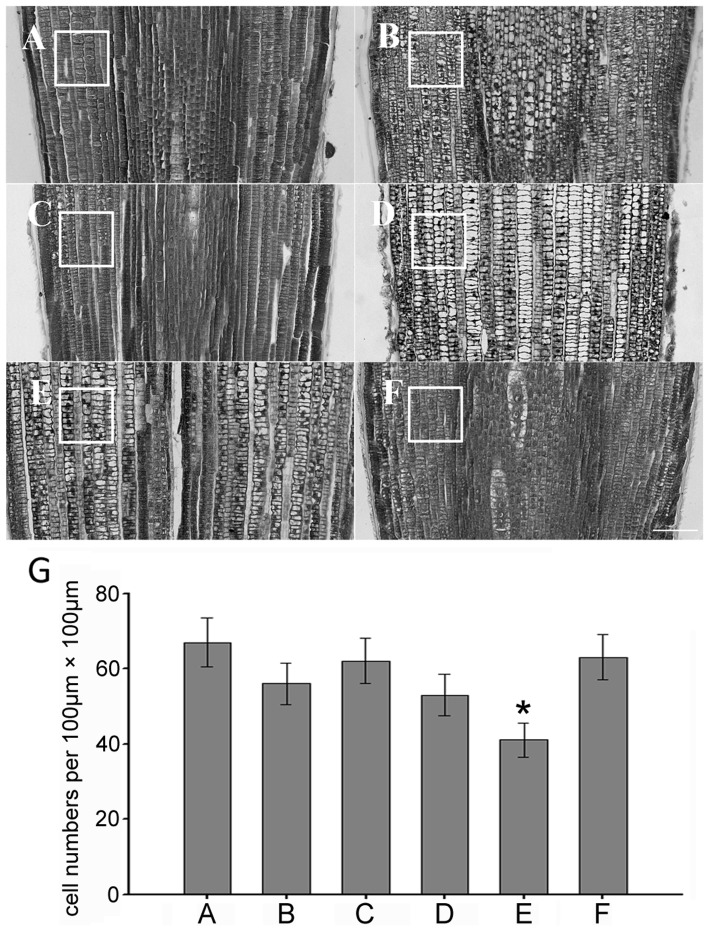
Histological analysis of root meristem cells. The images of paraffin wax sections of maize root meristem under different stresses are shown. (A) The control group. (B) CuSO_4_ treatment. (C) Mannitol treatment. (D) NaCl treatment. (E) Heat treatment. (F) Cold treatment. (G) Cell numbers per unit 100 µm×100 µm. Bars = 100 µm. * *p*<0.05 as compared with control group.

The effect of various abiotic stresses on the growth of maize seedling roots was investigated by comparing plants grown under control conditions and plants exposed to cold, heat, drought, high salinity and heavy metal stresses (Fig. S1). The strength or concentration of these stresses was based on the reported data and these treatments did not cause maize seedlings premature senescence. The primary root length, chlorophyll content, fresh weight and dry weight were assessed after treatment. The results showed that five different treatments all caused the decline of primary root length, fresh weight and dry weight compared with the control group. The primary root length was reduced by 30%–45% in response to abiotic stresses (Fig. 1A), while fresh weight and dry weight were respectively reduced to 43%–68% and 45%–78% (Fig. 1B and C). The leaf chlorophyll content under abiotic stresses but cold stress revealed a substantial rise: the content of chlorophyll *a* was elevated by about 2.5-fold and the content of chlorophyll *b* was elevated by 1-fold (Fig. 1D). To ascertain whether the histological pattern was affected, using tissue sections of paraffin wax embedding, the cell number and arrangement in the root meristem under different treatments for 24h were examined. The results showed that the cell number, size and arrangement were affected by CuSO_4_, NaCl, cold, mannitol and heat treatments compared with the control group (Fig. 2; Fig. S2).

### Various abiotic stresses increase the histone acetylation level

**Figure 3:**
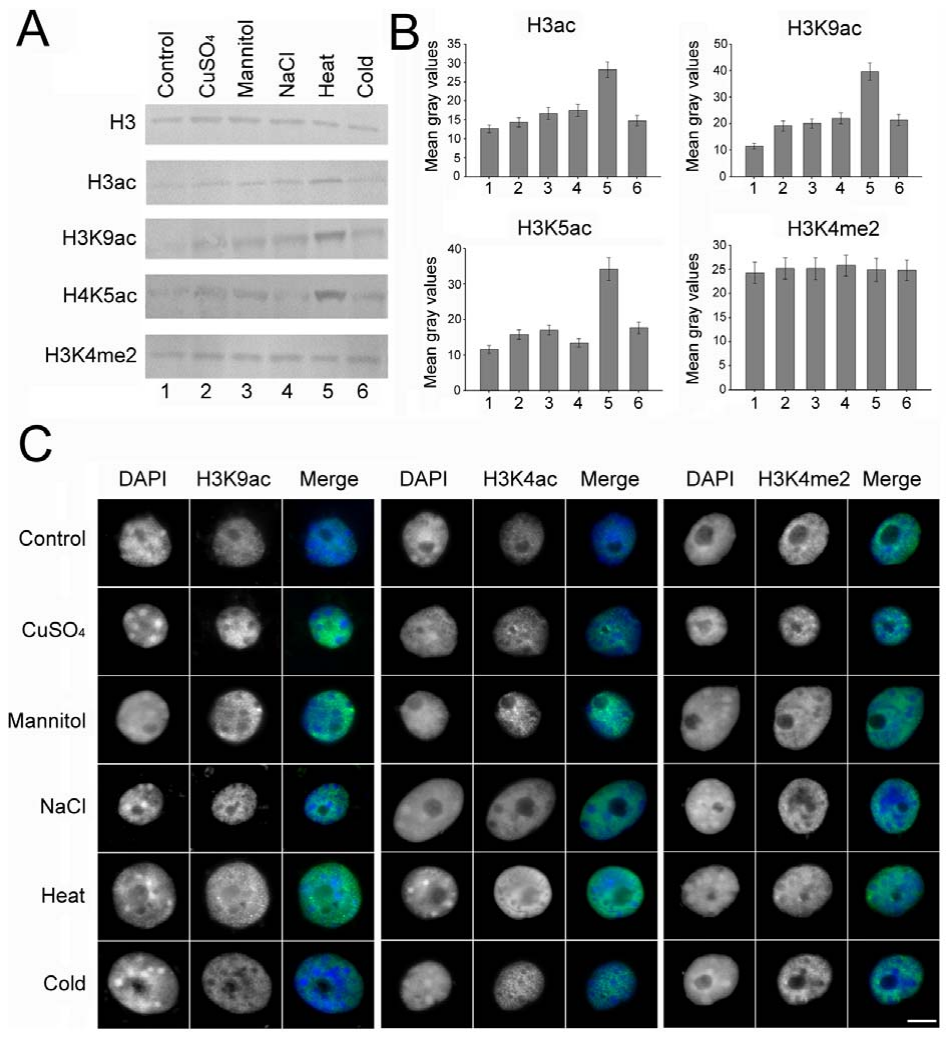
Different treatments affect global levels of histone modification. (A) Western blot was performed using H3, H3ac, H3k9ac, H4K5ac and H3K4me2 antibodies in roots of maize seedlings exposed to different treatments. (B) Histogram showing the mean gray values of the western blot bands for histone modification. Error bars represent the standard error of the mean. The experiments were repeated three times using biologically independent samples. (C) Nuclei from seedlings roots under different treatments for 24h were immunostained (H3K9ac, H4K5ac and H3K4me2 panels) and counterstained with DAPI (DAPI panel). The ‘Merge’ panel shows a merged image of blue and green staining. More than 200 nuclei were analyzed for every antibody. Bar = 10 µm.

Modification of lysine residues on histone tails has been correlated with gene expression in response to the environmental stresses. Several histone modifications are dramatically altered on the stress-responsive gene regions under abiotic stress conditions [30]. To ascertain the alterative pattern of histone modification in response to abiotic stress, Western blot analysis with proteins and in situ immunostaining analysis with interphase nuclei from normal and treated maize seedlings roots were performed. The results showed that the total acetylation levels under various treatments in the roots of maize seedlings were increased compared with the control group. Interestingly, this elevation was especially remarkable under heat stress (Fig. 3). The signals of H3K9ac and H4k5ac, regarded as positive histone modification marks associated with gene activation, were evenly dispersed in the nucleus, but the nucleoli and heterochromatin knobs were barely acetylated (Fig. 3C). For the methylation, there is no obvious change on the H3K4me2 level in both roots.

### Exogenous stress blocks cell cycle progression

**Figure 4:**
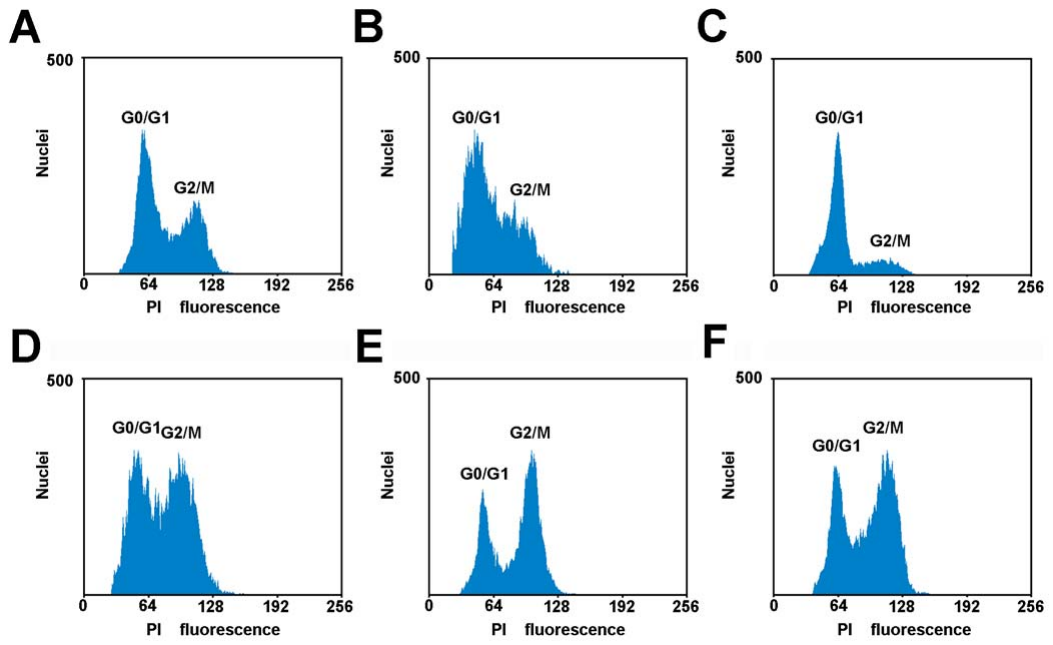
Flow cytometric analysis of cell cycle under the different types of stress. (A) The control group. (B) CuSO_4_ treatment. (C) Mannitol treatment. (D) NaCl treatment. (E) Heat treatment. (F) Cold treatment. All samples were treated for 24 h and stained with PI.

It has been reported that cold impairs cell cycle progression in maize. Therefore, to investigate whether the various types of abiotic stress inhibit cell cycle progression during a specific cell cycle phase, the nuclear DNA content was analyzed by flow cytometry. The results showed that abiotic stresses induced changes in cell cycle progression. Mannitol treatment caused most of the cells to be blocked in G1 phase because of the occurrence of the single 2C peak in the flow fluorescence graph. Heat and cold treatments both induced a pronounced cell accumulation in G2/M while CuSO_4_ treatment might induce cells not to go through S phase smoothly. The treatment with NaCl results in an extensive inhibition in both S and G2/M phase (Fig 4). These results imply that abiotic stresses lead to a longer time for cells to progress through the cell cycle. However, each treatment affects cell cycle in a different manner.

### Abiotic stresses regulate cell cycle gene expression

**Figure 5:**
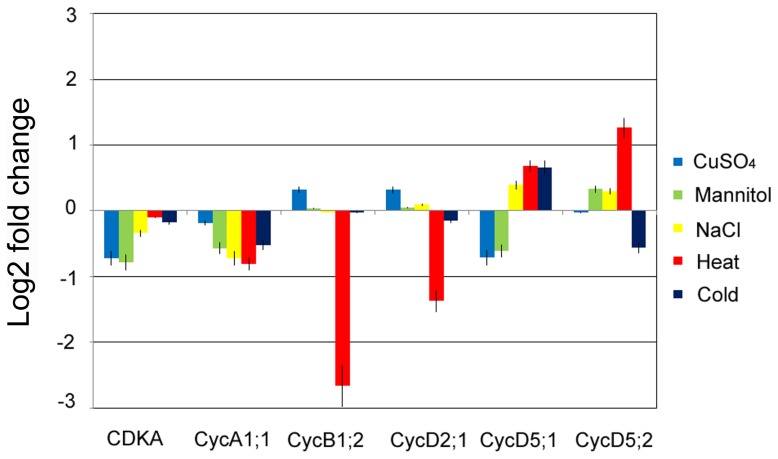
Expression change of *CDKA*, *CycA1;1*, *CycB1;2*, *CycD2;1*, *CycD5;1* and *CycD5;2* genes under abiotic stress. RNA samples were extracted from the control and treatment groups for 24 h. Compared with gene expression of the control group by real-time PCR, fold change form averages of three biological replicates are shown.

Cold nights affect cell cycle progression through transcriptional changes of cell cycle genes [2]. CDK is a cyclin-dependent kinase. To identify the changes in the molecular regulatory machinery of cell cycle, the expression of *CDKA* and five identified cell cycle genes (*CycA1;1*, *CycB1;2*, *CycD2;1*, *CycD5;1* and *CycD5;2*) in maize seedling roots was determined by real-time PCR using three independent biological repeats (Fig 5). The expression of *CDKA* and *CycA1;1* both were decreased in response to different stresses, whereas other cell cycle genes were differentially expressed under the adverse conditions. Among all treatments, heat stress exhibited a significant effect on expression level of these genes. Taken together, these expression data indicated that a slower cell cycle progression in roots grown under abiotic stresses was associated with positive and negative transcriptional changes of some cell cycle genes. These results suggested that transcriptional regulation of cell cycle gene plays an important role in the decreased cell multiplication and growth in response to abiotic stresses.

### Chromatin immunoprecipitation (ChIP) Analysis

**Figure 6:**
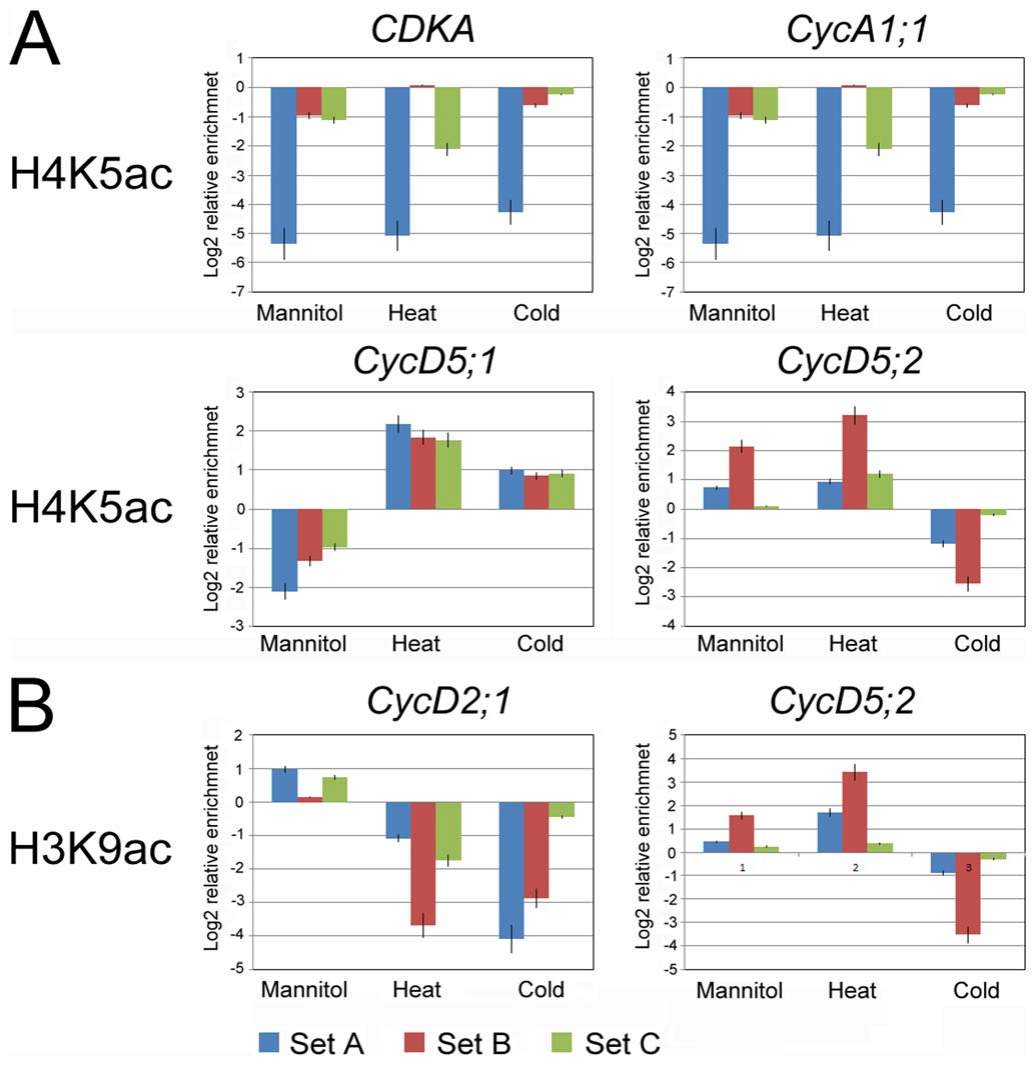
Alteration of H3K9ac and H4K5ac on the promoter of *CDKA*, *CycA1;1*, *CycB1;2*, *CycD2;1*, *CycD5;1* and *CycD5;2* genes. Graphs indicate the relative enrichment of functional histone modifications at the promoter **regions** (**Set A–C**) of genes in untreated root cells and after 24 h of different treatments analyzed by ChIP experiments. (A) Alteration of H4K5ac on the promoter region of *CDKA*, *CycA1;1*, *CycD5;1* and *CycD5;2* genes was consistent with the alteration of expression. (B) Alteration of H3K9ac on the promoter region of *CycD2;1* and *CycD5;2* genes was consistent with the alteration of expression. The standard errors are calculated from three independent ChIP assays and three real-time PCR reactions for each assay.

The promoter-associated dynamic histone acetylation/deacetylation provides a general mechanism for reversible gene regulation in response to environmental cues [31], [32]. The expression analysis indicated that abiotic stresses changed transcriptional responses at a subset of cell cycle genes. To determine whether transcriptional activation and repression was correlated with dynamic changes in histone H3 and H4 modification at the promoters of cell cycle genes, chromatin immunoprecipitation using H3ac, H3K9ac and H4K5ac antibodies was performed (Fig. S3; Fig. S4). Enrichment of H3K9ac and H4K5ac are positively correlated with gene activation of stress-responsive [22]. H3ac is histone 3 whose acetylation occurs at several different lysine positions in the histone H3 N-tail and was used as an overall control. In response to abiotic stresses, the alteration of H3K9ac enrichment on the promoter regions of *CycD2;1* and *CycD5;2* was consistent with the alteration of expression, and the alteration of H4K5ac enrichment on the promoter regions of *CDKA*, *CycA1;1*, *CycD5;1* and *CycD5;2* was consistent with the alteration of expression (Fig. 6). These results indicated that H3K9ac may be involved in the transcriptional regulation of *CycD2;1* and *CycD5;2*, and H4K5ac is likely to be associated with the transcriptional regulation of *CDKA*, *CycA1;1* and *CycD5;1*. The promoter region of *CycD5;2* occurred enrichment of H3ac, H3K9ac and H4K5ac in response to stresses. *CycB1;2* might be regulated by other modification sites in the histone N-tails.

## Discussion

In this study, the effect of various types of external stresses on plant growth and response was investigated at morphological, histological, cellular, molecular and epigenetic levels. The results provide evidence that abiotic stresses induce dynamic histone acetylation change which participates in the control of cell cycle gene transcription, thus resulting in perturbation of cell cycle progression and an inhibitory effect on growth and development in maize roots.

### Abiotic stress inhibits root growth rate by disturbing cell cycle progression

Adverse environmental stresses display the negative impact on the organism in a variety of ways. Under abiotic stress conditions, the root growth is inhibited and the cell division and cell cycle regulation are involved in this inhibition [9]. The data reveal that abiotic stresses have a strong inhibitory effect on maize seedling growth and development. The cell numbers per area in the root meristem are reduced and the arrangement becomes loose under abiotic stress as shown by histology analysis by paraffin wax sections. Further flow cytometry analysis reveals that the roots growth inhibition by abiotic stresses is tightly associated with the reduction of cell production. Therefore this reduction by abiotic stresses is a consequence of prolonged cell cycle duration but not of a reduced cell number or a smaller cell size in the root meristem. The flowcytometry data in our study indicate that G2 phase cells are more sensitive to extreme temperature, while osmotic stresses act specifically on the G1-S transition or S-G2 transition. Progression through its different phases (G1, S, G2, and M) is controlled by a class of heterodimeric protein kinases composed of a catalytic subunit, CDK and a regulatory subunit, cyclin. At the G1-S transition, CDKA forms a complex with D-type cyclins. While A- type and B-type cyclins expressed during the S-G2 and G2-M phases [33]. CDKs (CDKA) is constitutively expressed during the cell cycle and is required for cell cycle progression, but its expression is decreased in response to abiotic stress and therefore cell cycle progression could be blocked. At the G1-S transition, CDKA forms a complex with D-type cyclins, and A-type and B-type cyclins are expressed during the S-G2 and G2-M phases. Mannitol treatment changed the expression of D-type cyclin, probably leading to cell cycle arrested in G1 phase. Heat and cold treatments both induced a cell cycle arrested in G2/M, which might be related to a decrease in B-type cyclin (*CycB1;2*) expression. CuSO4 caused block of most cells in S phase was correlated with a reduction of A-type cyclin (*CycA1;1*) expression. The transcriptional regulation of the cell cycle gene in maize roots plays an important role in cell cycle arrest in response to abiotic stresses.

### Histone acetylation correlates with cell cycle gene transcriptional regulation in response to abiotic stresses

Acetylated histones are usually associated with transcriptionally active chromatin and deacetylated histones with inactive chromatin [34], [35]. The response of global acetylation and deacetylation are thought to allow for rapidly restoring acetylation levels after the removal of stresses, which indicates that maintenance of a proper acetylation state is an important aspect of chromatin dynamics [36], [37]. Analysis of nucleosomal response in maize seedlings revealed that maize core histones also undergo an genome-wide increase in the global level of histone acetylation in various degrees in response to abiotic stresses. The altered states of histone acetylation is not only likely to be important for processes occurring on chromatin, such as recombination, DNA repair, and replication, but also tightly correlated with transcriptional regulation [36], [38], [39]. In this study, the increased acetylation of the maize genome is likely to have analogous function in abiotic stress tolerance.

**Figure 7:**
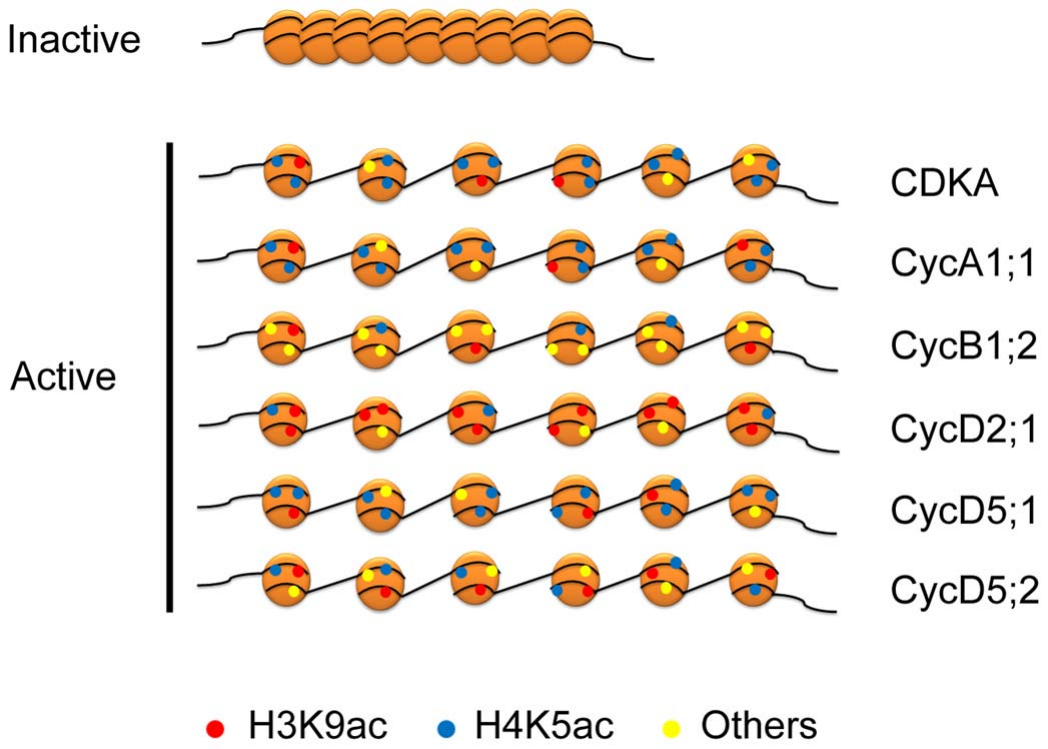
Schematic models for dynamic histone modification in *CDKA*, *CycA1;1*, *CycB1;2*, *CycD2;1*, *CycD5;1* and *CycD5;2* genes in response to abiotic stresses. The enrichment of histone modifications of H3K9ac (red circle), H4K5ac (blue circle), other (yellow circle) are indicated under abiotic stresses.

Distinct patterns of cell cycle gene expression play an important role in cell cycle control [40]. The analysis of cell cycle gene expression in maize roots provides an insight into potential mechanisms that regulate the cell cycle progression in response to abiotic stresses. The results show that transcriptional regulation of cell cycle gene expression plays a key role in the perturbation of cell cycle progression and growth inhibition during abiotic stresses. Recent research has highlighted the role of histone modification in the control of genes transcription. Histone modifications of chromatin on the promoters can be used for the storage of information about developmental and environmental cues [41]. At the single gene level, dynamic alteration of modifications in the N-tails of histones H3 and H4 is associated with transcriptional regulation of cell cycle genes in response to abiotic stresses. Each core histone has multiple sites at which modifications are found. However, most single histone proteins are not modified at every site. Some modification sites are therefore favoured over others and, even for a single histone, there can be many permutations of modification-site usage [42]. A combination of modified and unmodified acetylation sites at the promoter of a gene could lead to transcriptional activation [43]. *CDKA*, *CycA1;1*, *CycB1;2*, *CycD2;1*, *CycD5;1 and CycD5;2* showed their different patterns of histone acetylation in the abiotic stress responses (Fig. 7). These results indicated that enrichment of H3K9ac may function in transcriptional regulation of *CycD2;1*, and enrichment of H4K5ac may function in transcriptional regulation of *CDKA*, *CycA1;1* and *CycD5;1*. H3K9ac and H4K5ac may regulate the expression of *CycD5;2* together. H3K9ac and H4k5ac are not enriched on the promoter region of *CycB1;2*, suggesting that other modified sites maybe play an important role in transcriptional regulation under abiotic stresses. This stress-mediated histone acetylation is selective and targeted to specific gene, but this selection mechanism remains unknown. However, these data provide important information that histone modification is widely associated with the transcriptional regulation of cell cycle genes.

## Supporting Information

Figure S1 **The vitality of maize roots under different conditions.** All the samples are stained by 1-Naphthylamine. The darker-staining of root indicates the greater vitality. (A) The control group. (B) CuSO_4_ treatment. (C) Mannitol treatment. (D) NaCl treatment. (E) Heat treatment. (F) Cold treatment.
(TIF)

Figure S2 **The transverse analysis of maize roots under different conditions.** Transverse sections show the histological feature under normal and treatment conditions. (A) The control group. (B) CuSO_4_ treatment. (C) Mannitol treatment. (D) NaCl treatment. (E) Heat treatment. (F) Cold treatment. Bars = 200 µm
(TIF)

Figure S3 **Schematic representation of the primer sets within the promoter regions (A–C) of the cell cycle genes.**
(TIF)

Figure S4 **Alteration of H3ac, H3K9ac and H4K5ac on the promoter regions of** ***CDKA*** **,** ***CycA1;1*** **,** ***CycB1;2*** **,** ***CycD2;1*** **,** ***CycD5;1*** **and** ***CycD5;2*** **genes.** Graphs indicate the relative enrichment of histone modifications at the promoter regions (Set A–C) of genes in untreated root cells and after 24 h of different treatments analyzed by ChIP experiments. The standard errors are calculated from three independent ChIP assays and three real-time PCR reactions for each assay.
(TIF)

Table S1 **Primer sequences used for quantitative real-time PCR.**
(JPG)

Table S2 **Primer sequences used for ChIP-PCR.**
(JPG)
