# Supplementary figures and images for: Transcriptional Regulation of Cell Cycle Genes in Response to Abiotic Stresses Correlates with Dynamic Changes in Histone Modifications in Maize
Source: PLoS One. 2014 Aug 29;9(8):e106070. doi: 10.1371/journal.pone.0106070 (PMC4149478; doi:10.1371/journal.pone.0106070)

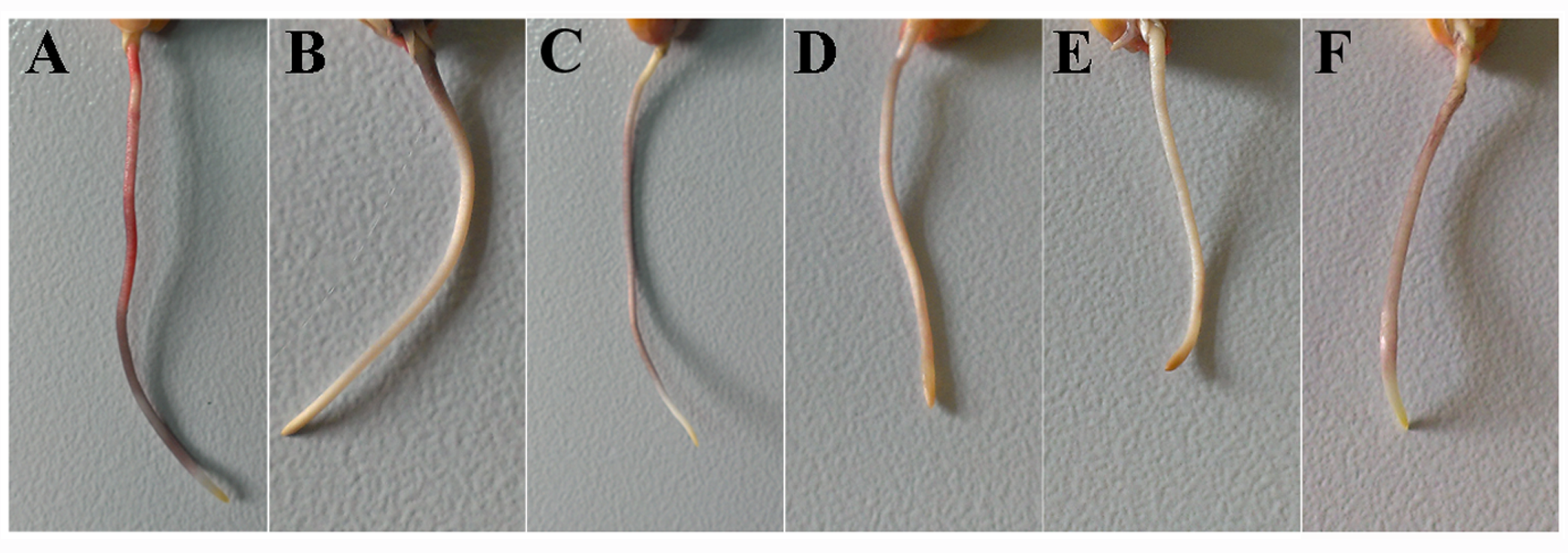

Supplement: Figure S1 — The vitality of maize roots under different conditions. All the samples are stained by 1-Naphthylamine. The darker-staining of root indicates the greater vitality. (A) The control group. (B) CuSO4 treatment. (C) Mannitol treatment. (D) NaCl treatment. (E) Heat treatment. (F) Cold treatment. (TIF) [file pone.0106070.s001.tif]

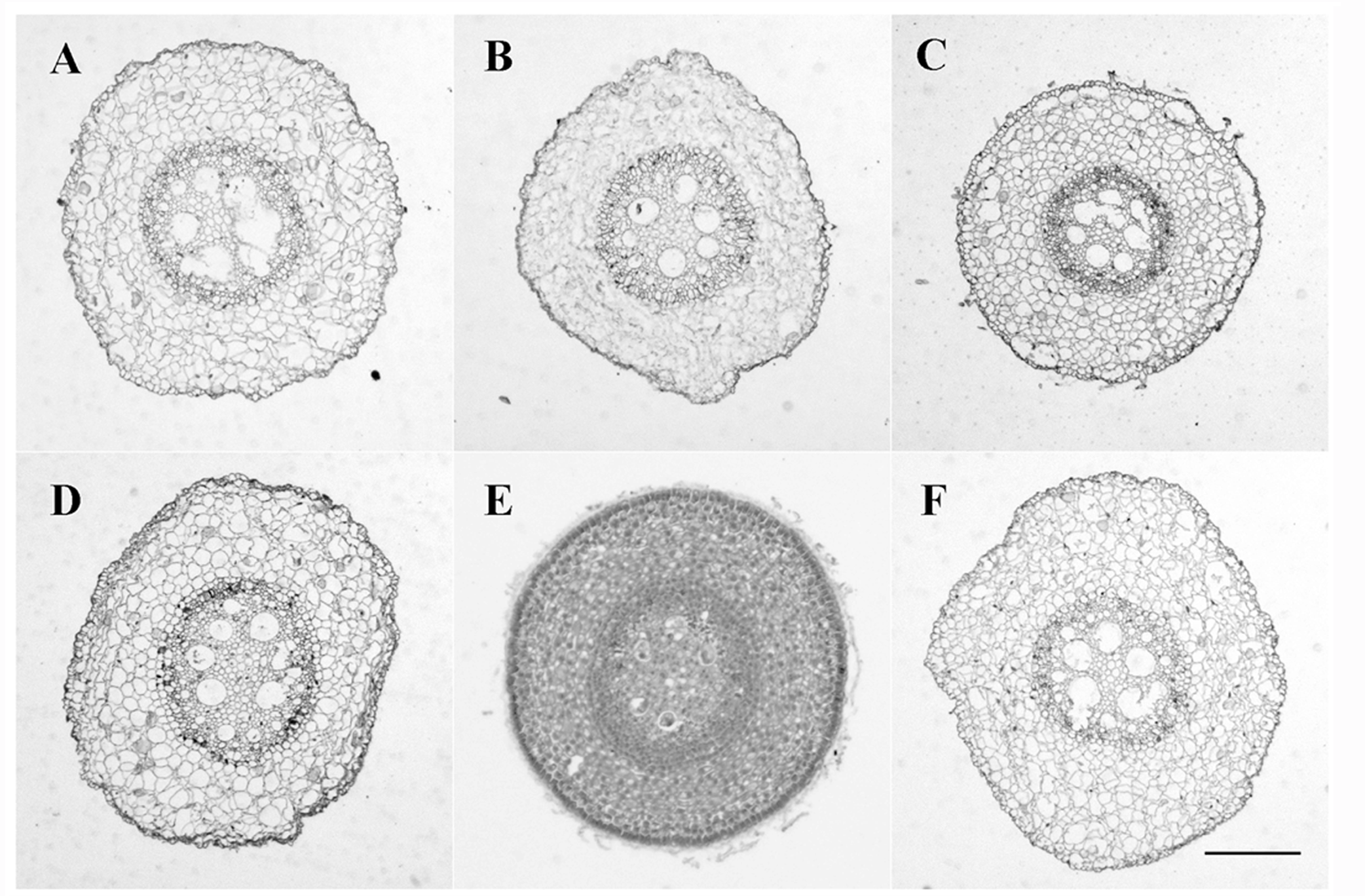

Supplement: Figure S2 — The transverse analysis of maize roots under different conditions. Transverse sections show the histological feature under normal and treatment conditions. (A) The control group. (B) CuSO4 treatment. (C) Mannitol treatment. (D) NaCl treatment. (E) Heat treatment. (F) Cold treatment. Bars = 200 µm (TIF) [file pone.0106070.s002.tif]

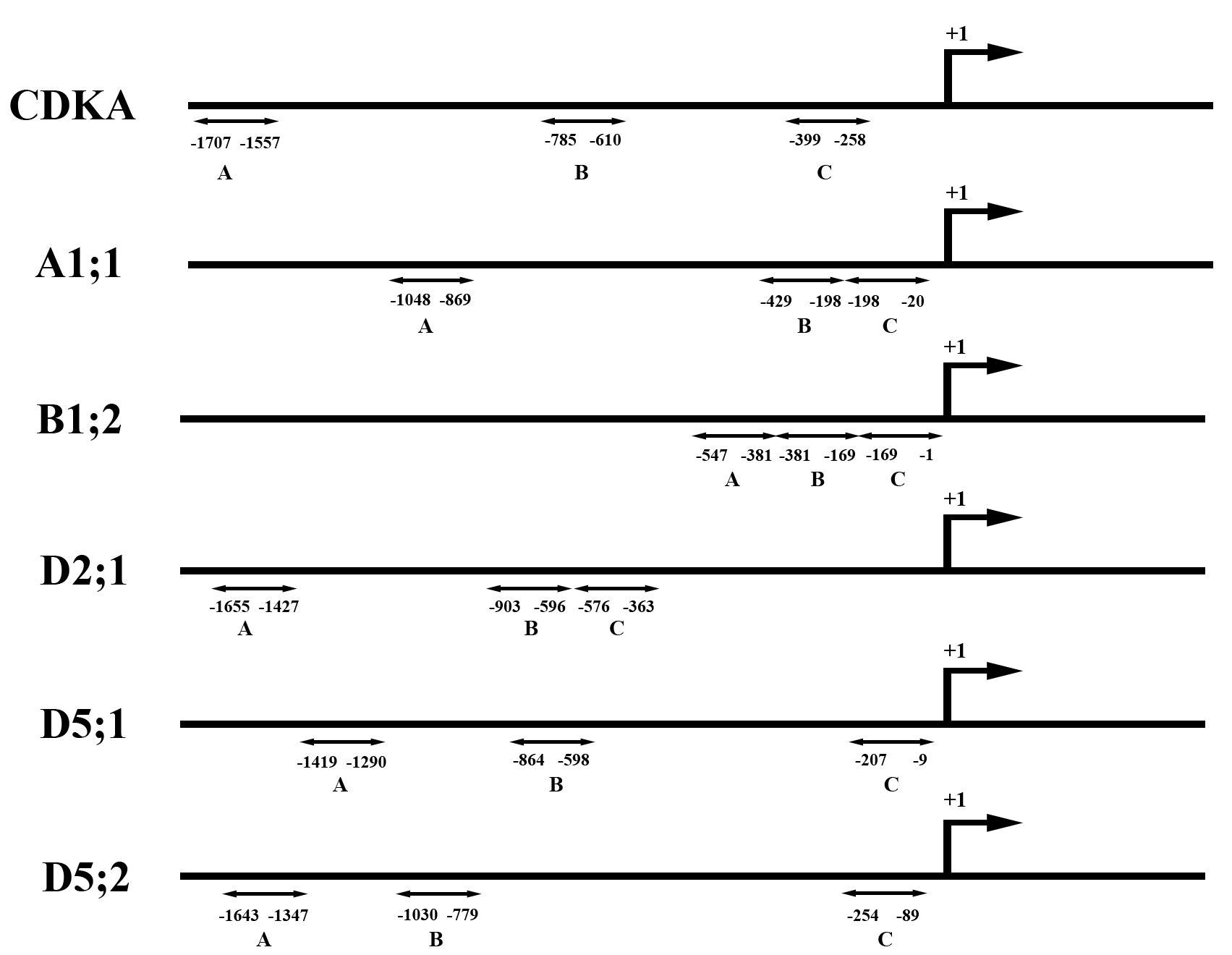

Supplement: Figure S3 — Schematic representation of the primer sets within the promoter regions (A–C) of the cell cycle genes. (TIF) [file pone.0106070.s003.tif]

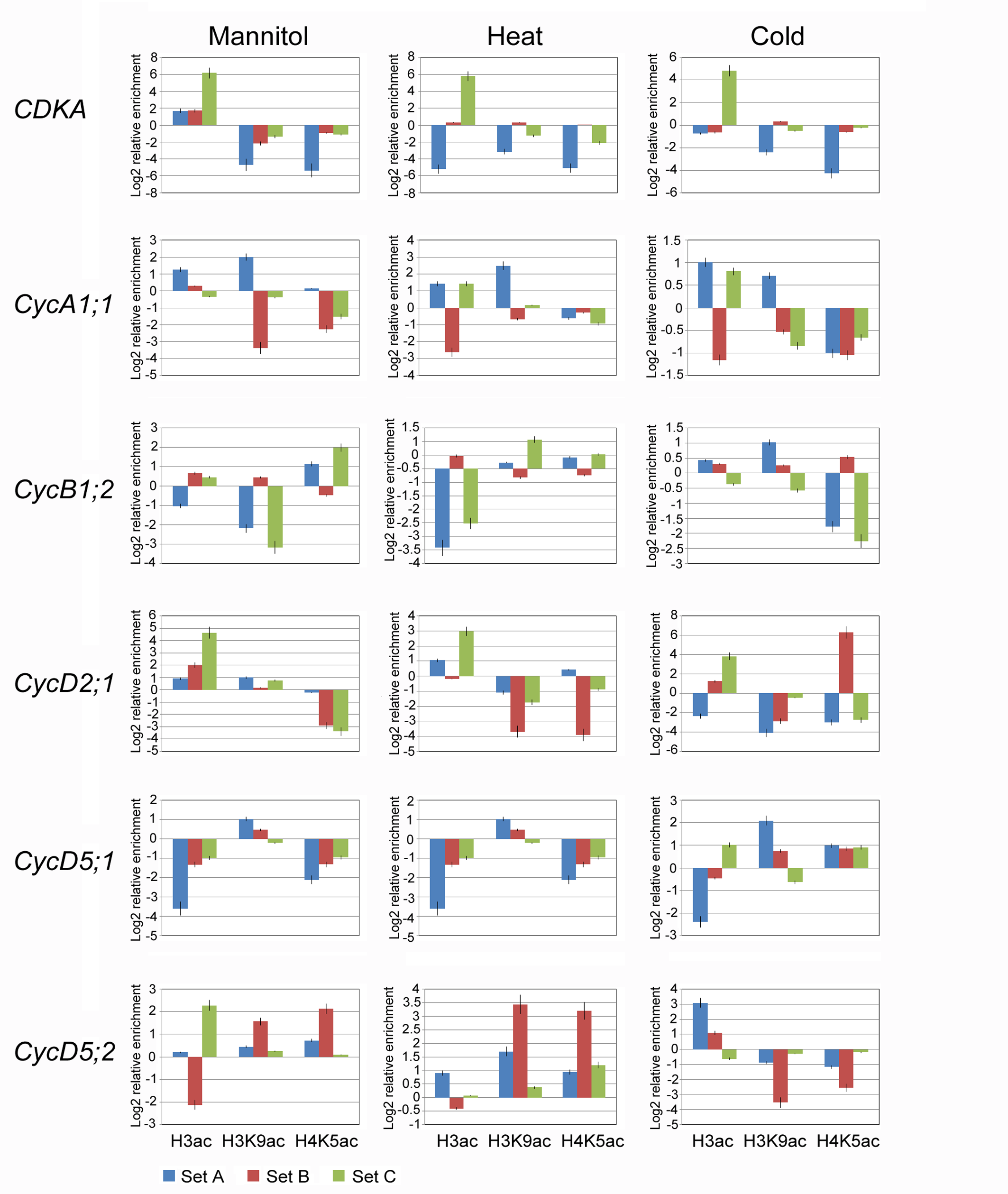

Supplement: Figure S4 — Alteration of H3ac, H3K9ac and H4K5ac on the promoter regions of CDKA , CycA1;1 , CycB1;2 , CycD2;1 , CycD5;1 and CycD5;2 genes. Graphs indicate the relative enrichment of histone modifications at the promoter regions (Set A–C) of genes in untreated root cells and after 24 h of different treatments analyzed by ChIP experiments. The standard errors are calculated from three independent ChIP assays and three real-time PCR reactions for each assay. (TIF) [file pone.0106070.s004.tif]

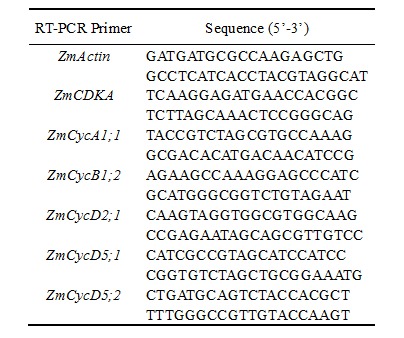

Supplement: Table S1 — Primer sequences used for quantitative real-time PCR. (JPG) [file pone.0106070.s005.jpg]

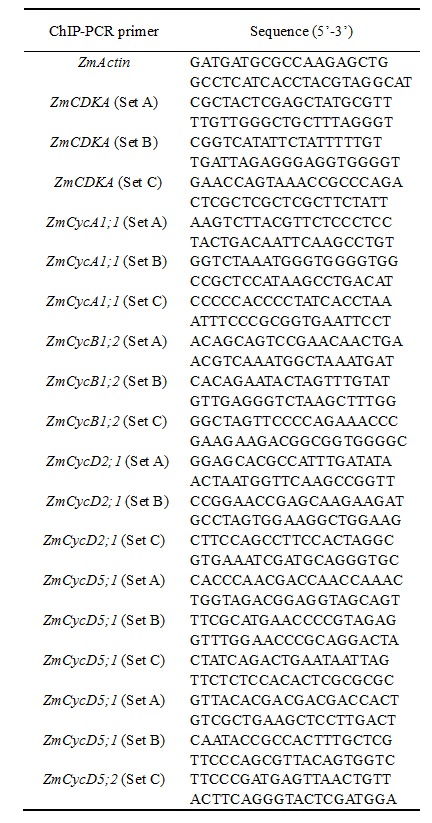

Supplement: Table S2 — Primer sequences used for ChIP-PCR. (JPG) [file pone.0106070.s006.jpg]
